# Prevalence of Diabetes Mellitus in People Living With HIV in Dammam, Saudi Arabia

**DOI:** 10.7759/cureus.63809

**Published:** 2024-07-04

**Authors:** Ali H Alsaeed, Ali H Aljanobe, Shaikha H Alhassan, Mohammed S Almulaify, Abdullah A AlKhalaf, Mousa J Alhaddad

**Affiliations:** 1 Infectious Disease, Dammam Medical Complex, Dammam, SAU; 2 Medicine, Medical University of Warsaw, Warsaw, POL; 3 Internal Medicine, Eye Specialist Hospital, Dhahran, SAU; 4 Internal Medicine, Dammam Medical Complex, Dammam, SAU; 5 Adult Hematology and Hematopoietic Stem Cell Transplantation, King Fahad Specialist Hospital, Dammam, SAU

**Keywords:** saudi arabia, hiv, hemoglobin a1c, prediabetes, diabetes mellitus

## Abstract

Backgrounds

The incidence of diabetes mellitus (DM) in people living with human immunodeficiency virus (HIV) receiving highly active antiretroviral therapy (HAART) is thought to be higher than that in noninfected people. The aim of this study was to investigate the prevalence of DM among people living with HIV in Dammam, Saudi Arabia (SA).

Methods

This was a cross-sectional study that included adult patients with HIV who were followed at Dammam Medical Complex. The electronic medical records of the patients were reviewed for their demographic data, comorbid conditions, and HIV history (e.g., duration and medications). The patients were categorized based on their glycated hemoglobin (A1C) levels into nondiabetic patients (A1C < 5.7%), prediabetic patients (A1C between 5.7% and 6.4%), and diabetic patients (A1C ≥ 6.5).

Results

A total of 769 HIV patients were assessed. The A1C of 325 patients could not be retrieved. The remaining 444 patients were included in the analysis. These consisted of 71 female patients (15.99%) and 373 male patients (84.01%). The average age of the patients was 38.62±11.33 years. Their duration for living with HIV was on average 3.76±3.15 years. The cohort consisted of 290 nondiabetic patients (65.32%), 107 prediabetic patients (24.1%), and 47 diabetic patients (10.59%). The nondiabetic patients were generally younger than the prediabetic patients (35.97 vs 40.72 years on average, P value < 0.001). They were infected with HIV for shorter durations (3.45 vs 4.19 years on average, P value < 0.05) with a higher percentage of patients receiving antiretroviral therapy (97.93% vs 84.11%, P value < 0.001). Similarly, the nondiabetic patients were generally younger than the diabetic patients (35.97 vs 50.19 years on average, P value < 0.001). They were also infected with HIV for shorter durations (3.45 vs 4.65 years on average, P value < 0.05) with, also, a higher percentage of patients receiving antiretroviral therapy (97.93% vs 89.36%, P value < 0.01).

Conclusions

The prevalence of DM among people living with HIV in Dammam, SA, was high with DM remaining highly underdiagnosed in this population. However, the prevalence of DM in this study involving mostly HIV patients treated with newer HAART agents was lower than what was reported in multiple previous studies that included patients using older agents.

## Introduction

It is estimated that more than 530 million people are affected by diabetes mellitus (DM) worldwide [1] with the highest rates being in the Middle East [1]. Specifically, Saudi Arabia (SA) has one of the highest age-standardized prevalences of DM at 11.3%, a figure that is almost double the global age-standardized DM prevalence of 6.1% [1]. Moreover, it is projected that type 2 DM prevalence will increase by more than 100% in SA by 2025 [1].

The prolonged survival of treated human immunodeficiency virus (HIV) patients has allowed for an increased focus on the management of chronic conditions including DM in this population especially since there is growing evidence supporting an association between DM and HIV or its medications. The incidence of DM in HIV-infected patients receiving highly active antiretroviral therapy (HAART) is three to four times higher than that in non-infected patients [2,3]. Many mechanisms were hypothesized to explain this association, including down-regulation of glucose transporters [4], elevated circulating inflammatory cytokine levels [5], defective insulin signaling [4], and development of lipodystrophy [5]. Thus, the current guidelines recommend screening for DM in HIV patients at baseline and starting and changing HAART [6].

Thus, the main objective of this study was to investigate the prevalence of DM among people living with HIV in Dammam, SA.

## Materials and methods

The study was a cross-sectional chart review study. It included adult patients with HIV who were followed at Dammam Medical Complex, the main governmental HIV center in the Eastern Health Region in SA. Their electronic medical records were reviewed in the second half of 2023 for their demographic information (such as age and gender), comorbid conditions (such as hypertension), and HIV history (such as duration of infection and medications), in addition to their CD4 cell counts and their hemoglobin and creatinine levels. The records were also reviewed for the results of their glycated hemoglobin (A1C) level.

The patients were categorized into nondiabetic patients (A1C < 5.7%), prediabetic patients (A1C between 5.7% and 6.4%), and diabetic patients (A1C ≥ 6.5) [7].

To calculate the prevalence of DM, the number of diabetic patients was divided by the total number of patients included in the study.

The nondiabetic patients were compared with both the prediabetic patients and the diabetic patients separately looking for any association between the patients' diabetic statuses and the patients' demographics, comorbid conditions, personal HIV data, and laboratory results.

To analyze the collected data, the Python programming language version 3.7.6 (Python Software Foundation, Wilmington, DE) was used along with the SciPy library 1.4.1 (Enthought, Inc., Austin, TX) and Statsmodels module (v0.11.1, Python package).

Count and percentage were used to describe the categorical variables. The continuous variables were described using measures of central tendency (i.e., mean and median) and measures of dispersion (i.e., median and interquartile range) as appropriate. The chi-square test was used to compare the categorical variables, and the two-sample t-test was used to compare the continuous variables. A p-value less than 0.05 was considered statistically significant.

The study was approved and closely monitored by the Institutional Review Board (IRB) of Dammam Medical Complex (IM-14, June 22, 2023).

## Results

A total of 769 people living with HIV patients were assessed; however, the A1C data for 325 patients were not available, leaving 444 patients for analysis. Among these, there were 71 female patients (15.99%) and 373 male patients (84.01%). The male-to-female ratio was 5.25. The average age of the patients was 38.62±11.33 years. Their duration for living with HIV was on average 3.76±3.15 years. The median viral RNA load was found to be 20 copies/mL, with an interquartile range spanning from 0 to 133 copies/mL. As for the median CD4 count, it was measured at 647.24 cells/microL, with an interquartile range of 389.98-936.52 cells/microL. The majority of patients, 416 in total (93.69%), were receiving HIV medications, while 24 patients (5.41%) had a comorbidity of hypertension. The cohort consisted of 290 nondiabetic patients (65.32%), 107 prediabetic patients (24.1%), and 47 diabetic patients (10.59%). The patients' demographics are shown in Table 1.

**Table 1:** Patients' Demographics (n = 444)

Characteristic		n (%)
Age (Mean ± SD, years)		38.62±11.33
Gender	Male	373 (84.01%)
	Female	71 (15.99%)
HIV Duration (Mean ± SD, years)		3.76±3.15
CD4 Count (Mean ± SD, cells/microlitre)		695.12±413.42
HIV Medications	Bictegravir, Emtricitabine, and Tenofovir Alafenamide, n (%)	225 (50.68%)
	Dolutegravir, Emtricitabine and Tenofovir Alafenamide, n (%)	72 (16.22%)
	Darunavir Cobicistat, Emtricitabine and Tenofovir alafenamide, n (%)	40 (9.01%)
	Raltegravir, Emtricitabine and Tenofovir Alafenamide, n (%)	40 (9.01%)
	Abacavir, Dolutegravir, and Lamivudine, n (%)	35 (7.88%)
	Others, n (%)	4 (0.9%)
	None, n (%)	28 (6.31%)
Hypertension		24 (5.41%)
Diabetes Mellitus	Diabetic	290 (65.32%)
	Prediabetic	107 (24.1%)
	Non-diabetic	47 (10.59%)
Hemoglobin A1C (Mean ± SD, %)		5.7±1.3
Hemoglobin (Mean ± SD, mg/g/dl)		14.14±2.02
Creatinine (Mean ± SD, mg/dl)		1.01±0.52

The nondiabetic patients were generally younger than the prediabetic patients (35.97 vs 40.72 years on average, P value < 0.001). They were infected with HIV for shorter durations (3.45 vs 4.19 years on average, P value < 0.05) with a higher percentage of patients receiving antiretroviral therapy (97.93% vs 84.11%, P value < 0.001). Moreover, they had fewer hypertensive patients (1.38% vs 8.41%, P value < 0.01). The detailed comparisons between the nondiabetic patients and the prediabetic patients are shown in Table 2.

**Table 2:** Comparison Between the Nondiabetic Patients and the Prediabetic Patients (n = 397) * A P value of less than 0.05 was used to indicate statistical significance.

Characteristic	Nondiabetic Patients (n = 290)	Prediabetic Patients (n = 107)	P value
Hemoglobin A1C, mean ± SD (%)	5.16±0.36	5.93±0.22	0*
Age, mean ± SD (years)	35.97±9.85	40.72±11.1	0*
Male, count (%)	249 (85.86%)	87 (81.31%)	0.3373
Weight, mean ± SD (kg)	70.76±16.0	77.79±20.75	0.0004*
HIV Duration, mean ± SD (years)	3.45±3.02	4.19±3.22	0.0373*
CD4 Count, mean ± SD (cells/microlitre)	683.17±416.84	740.33±387.36	0.3722
On HIV Medications, count (%)	284 (97.93%)	90 (84.11%)	0*
Hypertension, count (%)	4 (1.38%)	9 (8.41%)	0.0015*
Hemoglobin, mean ± SD (g/dL)	14.33±1.97	13.9±2.01	0.0548
Creatinine, mean ± SD (mg/dL)	0.98±0.38	1.03±0.48	0.3563

Similarly, the nondiabetic patients were generally younger than the diabetic patients (35.97 vs 50.19 years on average, P value < 0.001). They were also infected with HIV for shorter durations (3.45 vs 4.65 years on average, P value < 0.05) with a higher percentage of patients receiving antiretroviral therapy (97.93% vs 89.36%, P value < 0.01). Moreover, they had fewer hypertensive patients (1.38% vs 23.4%, P value < 0.001). However, as opposed to the prediabetic patients, the diabetic patients had lower hemoglobin (13.59 vs 14.33 g/dL on average, P value < 0.05) and higher creatinine levels (1.19 vs 0.98 mg/dL on average, P value < 0.05) than the no diabetic patients. The detailed comparisons between the nondiabetic patients and the prediabetic patients are shown in Table 3.

**Table 3:** Comparison Between the Nondiabetic Patients and the Diabetic Patients (n = 337) * A P value of less than 0.05 was used to indicate statistical significance.

Characteristic	Nondiabetic Patients (n = 290)	Diabetic Patients (n = 47)	P value
Hemoglobin A1C, mean ± SD (%)	5.16±0.36	8.53±2.27	0*
Age, mean ± SD (years)	35.97±9.85	50.19±12.35	0*
Male, count (%)	249 (85.86%)	37 (78.72%)	0.2949
Weight, mean ± SD (kg)	70.76±16.0	82.97±21.42	0*
HIV Duration, mean ± SD (years)	3.45±3.02	4.65±3.52	0.0147*
CD4 Count, mean ± SD (cells/microlitre)	683.17±416.84	678.83±456.22	0.963
On HIV Medications, count (%)	284 (97.93%)	42 (89.36%)	0.0087*
Hypertension, count (%)	4 (1.38%)	11 (23.4%)	0*
Hemoglobin, mean ± SD (g/dl)	14.33±1.97	13.59±2.24	0.0207*
Creatinine, mean ± SD (mg/dl)	0.98±0.38	1.19±1.06	0.0154*

## Discussion

In this study, the diabetic patients were generally older than the prediabetic patients. The prediabetic patients were also older than the non-diabetic patients. This supports that age is an important risk factor for DM in people living with HIV as it is in the general population [8-10]. Our results also supported that the risk of DM in people living with HIV increases with the longer duration of HIV infection [11,12]. Moreover, DM in people living with HIV was shown both in this study and other studies to be associated with hypertension and renal impairment [11-13].

The prevalence of DM among people living with HIV in this study was 10.6%. It was lower than that of two previous studies in SA [14,15], which reported a prevalence of around 15.9%. However, it was not higher than what is reported in the general population in SA [1]. Thus, our results are not suggestive of an association between DM and HIV or its medications. On the opposite, the HIV patients who were receiving antiretroviral therapy were more likely to be nondiabetic rather than prediabetic or diabetic.

The lack of association between DM and HIV could be attributed to a variety of reasons. First, most patients in this study were using newer HAART agents. The original studies that suggested a link between DM and antiretroviral therapy evaluated patients using older antiretroviral agents, such as Stavudine and Indinavir [5,12,16-18]. Many other studies did not consistently demonstrate an increased risk of DM in patients receiving newer antiretroviral agents [18-24]. Secondly, the HIV population in this study was relatively young. Thus, the prevalence of DM would not be expected to be as high as in the general population. Thirdly, more than 80% of the included patients were men. The odds of having DM are higher in women with HIV than women without HIV, but men with HIV might not be different in their risk for developing DM from men without HIV [12,25,26]. Lastly, we used A1C as the screening test for DM. It is known that A1C might inaccurately underestimate blood glucose levels in patients with HIV [27-29] although this was not consistently proven [30].

The study had several limitations. The first is that the study had a cross-sectional design. A temporal relationship between HIV and the development of DM could not be established. Thus, some of the diabetic patients might have developed DM prior to the infection with HIV and the initiation of HAART. Secondly, the retrospective collection of data could have resulted in potential biases from the missing and inaccurately documented information. This was markedly evident as 315 patients were excluded for not having A1C values. Thirdly, the use of A1C as a screening test for DM might not be optimal in people living with HIV [27-29]. Fasting blood glucose is more recommended in this population [6]. Finally, the results of this study being conducted at a single center in Dammam Medical Complex might not be generalizable. The study, therefore, cannot represent, for example, the patients who choose - due to the HIV-related stigma - to not seek medical attention or to seek it in a non-governmental center [13].

## Conclusions

The prevalence of DM in people living with HIV is high; however, it remains uncertain whether HIV contributes to the development of DM. It is crucial for future studies to address the limitations of our current study by employing a multicenter prospective design, including larger samples, and incorporating screening modalities beyond A1C measurement.

Our findings supported that, among people living with HIV, diabetic individuals tended to be older, had a longer duration of HIV infection, lower adherence to antiretroviral therapy, lower hemoglobin levels, higher creatinine levels, and a higher prevalence of hypertension compared to nondiabetic patients. These observations underscore the significance of comprehensive care for HIV patients and their associated comorbidities.
